# Robust 3D modeling reveals spatiosyntenic properties of animal genomes

**DOI:** 10.1016/j.isci.2023.106136

**Published:** 2023-02-04

**Authors:** Tereza Clarence, Nicolas S.M. Robert, Fatih Sarigol, Xiao Fu, Paul A. Bates, Oleg Simakov

**Affiliations:** 1 Biomolecular Modelling Laboratory, The Francis Crick Institute, 1 Midland Road, London NW1 1AT, UK; 2 Department of Neuroscience and Developmental Biology, University of Vienna, Vienna, Austria; 3 Roussos Lab/Center for Disease Neurogenomics, Icahn School of Medicine at Mount Sinai, New York, NY, USA

**Keywords:** Genomics, Molecular biology, Evolutionary biology

## Abstract

Animal genomes are organized into chromosomes that are remarkably conserved in their gene content, forming distinct evolutionary units (synteny). Using versatile chromosomal modeling, we infer three-dimensional topology of genomes from representative clades spanning the earliest animal diversification. We apply a partitioning approach using interaction spheres to compensate for varying quality of topological data. Using comparative genomics approaches, we test whether syntenic signal at gene pair, local, and whole chromosomal scale is reflected in the reconstructed spatial organization. We identify evolutionarily conserved three-dimensional networks at all syntenic scales revealing novel evolutionarily maintained interactors associated with known conserved local gene linkages (such as hox). We thus present evidence for evolutionary constraints that are associated with three-, rather than just two-, dimensional animal genome organization, which we term spatiosynteny. As more accurate topological data become available, together with validation approaches, spatiosynteny may become relevant in understanding the functionality behind the observed conservation of animal chromosomes.

## Highlights

• Interaction sphere approach enables comparative spatial genomics
• Distantly related animal genomes show conserved spatial organization
• The approach helps reveal spatially co-localized genes across species (spatiosynteny)

## Graphical abstract

**Figure:**
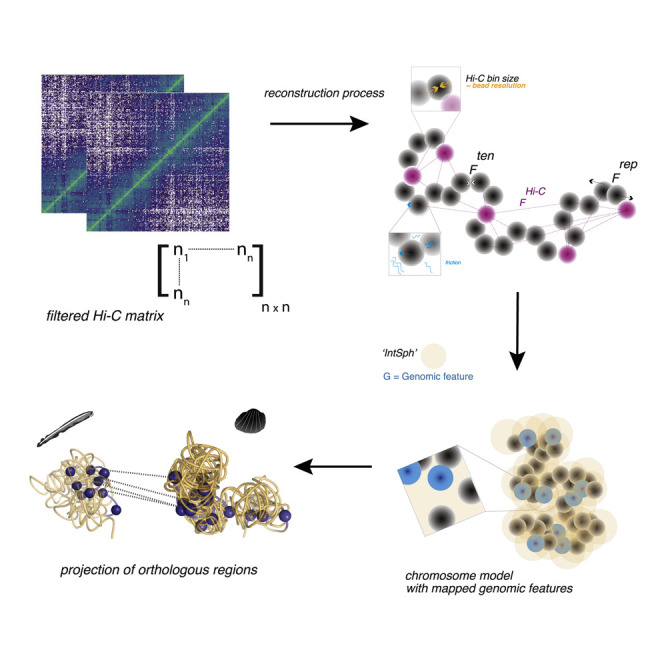

## Introduction

**Figure 1:**
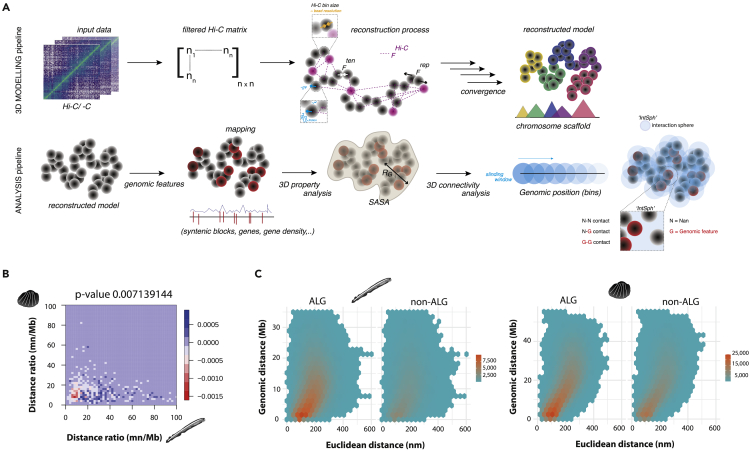
Hi-Chrom modeling pipeline and its utilization for macrosynteny interactions (A) Schematic of 3D modeling tools and analysis pipeline; first row depicts the workflow of Hi-C pre-processing and modeling. Second row shows mapping of genomic features and their consequent structural analysis. (B) Difference of normalized observed and randomized spatial ratios (nm distance to megabase distance) of orthologous gene pairs. Fasano Franceschini test p value is shown (median of 10 randomized orthology runs, Bonferonni-corrected). (C) Density hexplot depicting relationship between genomic and Euclidean distance of ALG or nonALG contact pairs for amphioxus and blood clam, respectively.

Gene order, defined solely based on the one-dimensional chromosomal location, is largely conserved at local (subchromosomal) and chromosomal genomic levels across vast evolutionary distances in animals (600 million years^1^^,^^2^^,^^3^)—termed micro- and macrosynteny, respectively. Previous reports show evidence for functional linkages of genes in microsyntenies.^1^^,^^4^^,^^5^ However, little is known about their three-dimensional (3D) organization across animals and whether there is any selective pressure to maintain the significantly conserved chromosomal organization (macrosynteny) across species.^6^ This observation of high conservation of macrosyntenic linkages in most animal species hints at some constraints, but any functional inference has been lacking due to the absence of chromosomal structural information.^7^ To begin testing this hypothesis, we describe a new versatile method, Hi-Chrom (Figure 1A), that utilizes available Hi-C information to reconstruct 3D models of chromosomes. We use it to measure 3D property of genes that constitute macro- and microsyntenic groups revealing that conventional synteny is reflected by contact-rich spatial organization, as well as revealing genomic regions that are “spatiosyntenic”, i.e., genomically distant but physically close.

## Results and discussion

### Modeling approach

Our approach utilizes a model consisting of beads on a string, with a diameter of each bead depending upon the resolution of input Hi-C data (Figure 1A). Beads are initially randomly dispersed and after multiple cycles imposed by the algorithm converge to stable chromosome topologies. During each cycle, beads slowly adjust their positions dependent upon simple physical forces in conjunction with the Hi-C constraints (Figure S1 and STAR Methods, Table 1, 2 and 3). To compensate for the highly variable—and often limited in the long-range contact information—Hi-C quality, we developed a partitioning approach where we subdivide the chromosomes into single interaction spheres (IntSphs, Figure 1A). IntSphs have uniform, user-defined, physical radii, helping enhance profiling of spatial intra-chromosome interactions within this restricted vicinity (Figure S2). Testing different IntSph sizes (Figure S2) allowed us to identify a set of radii that allow for consistent cross-species comparisons.

**Table 1:** Sequencing parameters from utilized Hi-C dataset

Short Name	Genome Size	Number of Paired Reads	Tissue	Read Length	Library Depth	accession ID
ANABR	884,566,040	174,148,156	muscle	150	59.0622344	NCBI SRA: SRX5337861
BRAFL	513,461,369	115,383,056	whole body	147	66.06654466	NCBI SRA: SRX3274438
DROME	143,726,002	88,682,876	embryo	100	123.4054726	NCBI SRA: SRX2947125
CAEEL	100,286,401	666,058,021	mixed stage embryos	101	1341.594861	NCBI SRA: SRX2638356
RHOES	256,689,583	203,573,974	whole body	150	237.9223632	NCBI SRA: SRX8210228
ACHFU	1,855,892,613	696,378,573	abdominal foot	150	112.5677049	NCBI SRA: SRX5181756
HUDVU	847,270,819	712,495,485	whole polyp	100	168.1860083	NCBI SRA: SRX14496554
PECMA	918,306,378	241,297,364	muscle	151	79.35456583	NCBI SRA: SRX6848914

**Table 2:** Hi-C constraints applied for single chromosome model reconstruction from mean/median and 35^th^ percentile IF threshold cutoff in amphioxus

Chromosomal scaffold	Mean IF	Median IF	35^th^ percentile	mean IF %	median IF %	35^th^ IF %
*Sc7u5tJ_339*	4.71	2	1.5	86	50.01	35
*Sc7u5tJ_1590*	4.58	1.98	1.47	85.76	50.01	35.01
*Sc7u5tJ_366*	4.79	1.99	1.48	86.56	50.01	35
*Sc7u5tJ_1568*	61.89	42.35	7.5	57.81	50	35.94
*Sc7u5tJ_1587*	5.94	2.37	1.83	86.58	50	34.99
*Sc7u5tJ_1571*	4.02	1.67	1.25	86.4	50	35
*Sc7u5tJ_1559*	3.83	1.58	1.17	85.79	50	35
*Sc7u5tJ_320*	6	2.26	1.71	88.29	50.01	35.01
*Sc7u5tJ_1517*	5.45	2.27	1.69	85.77	50	35
*Sc7u5tJ_350*	3.63	1.62	1.21	85.06	50	35
*Sc7u5tJ_1565*	7.11	2.5	1.89	89.12	50.01	35.01
*Sc7u5tJ_566*	5.46	2.12	1.58	87.64	50	35
*Sc7u5tJ_1552*	5	2.16	1.63	85.84	50	35.01
*Sc7u5tJ_1442*	3.48	1.55	1.16	84.24	50	35
*Sc7u5tJ_1579*	5.05	2.25	1.72	85.58	50	35
*Sc7u5tJ_190*	4.99	2.05	1.55	86.93	50	35
*Sc7u5tJ_1485*	5.75	2.45	1.87	86.56	50.01	35
*Sc7u5tJ_1398*	4.5	1.71	1.29	87.71	50	35
*Sc7u5tJ_417*	5.33	2.2	1.67	86.18	50	35

**Table 3:** Hi-C constraints applied for single chromosome model reconstruction from mean/median and 35^th^ percentile IF threshold cutoff in blood clam

Chromosomal scaffold	Mean IF	Median IF	35^th^ percentile	Mean IF %	Median IF %	35^th^ IF %
*Lachesis_group0*	7.45	3.02	2.04	78.26	50	35
*Lachesis_group1*	8.39	3.28	2.18	78.07	50	35
*Lachesis_group2*	8.31	3.27	2.13	77.65	50	35
*Lachesis_group3*	9.07	3.42	2.22	77.63	50	35
*Lachesis_group4*	8.17	3.51	2.41	77.23	50	35
*Lachesis_group5*	9.2	3.89	2.62	77.12	50	35
*Lachesis_group6*	8.75	3.77	2.58	78.44	50	35
*Lachesis_group7*	9.67	4.26	2.81	76.48	50	35
*Lachesis_group8*	9.58	4.06	2.68	77.06	50	35
*Lachesis_group9*	9.59	4.15	2.73	77.51	50	35
*Lachesis_group10*	9.56	4.1	2.67	77.47	50	35
*Lachesis_group11*	9.81	4.08	2.61	76.82	50	35
*Lachesis_group12*	9.96	3.84	2.49	76.73	50	35
*Lachesis_group13*	10.39	4.29	2.82	78.13	50	35
*Lachesis_group14*	11.32	4.83	3.17	76.8	50	35
*Lachesis_group15*	11.79	5.13	3.45	78.08	50	35
*Lachesis_group16*	12.9	5.55	3.73	78.02	50	35
*Lachesis_group17*	12.82	6.03	4.24	78.53	50	35
*Lachesis_group18*	14.42	6.68	4.74	79.12	50	35

In this study, we assessed chromosome structures and evolutionary conservation of several evolutionarily distant animal genomes: the chordate *Branchiostoma floridae*, the scallop *Pecten maximus*, the snail *Achatina fulica*, the nematode *Caenorhabditis elegans*, the fruit fly *Drosophila melanogaster*, the jellyfish *Rhopilema esculentum*, and the cnidarian *Hydra vulgaris* (Table 1). These species span the ancient origin and earliest diversification of animals (over 500 million years ago,^8^ Figures S3 and S6). We have validated models produced with our Hi-Chrom method against other prediction tools (LorGD,^9^ 3DMax,^10^ Figure S1, and Hi-C filtering approaches such as FitHiC^11^⁠) showing similar predicted topologies. While most of the currently available methods, including ours, agree on the overall structure, many of the key phylogenetic clades have only limited Hi-C studies available that lack tissue or cell-type-resolved topological information. The availability of the IntSph profiling is thus crucial for any comparative study. Implementation of the IntSph approach in other tools, along with independent experimental validation, may provide for further comparative possibility and further elaboration of the results presented here.

### Syntenic conservation in spatial organization

With the chromosomal models and gene orthology information (STAR Methods), we have asked whether any of the orthologous co-localization at gene pair, macro- and microsyntenic levels are reflected in the three-dimensional structures. For this, we measured the Euclidean (3D, nanometer, nm) and genomic (1D, megabase pair, Mb) distances between orthologous bins along the chromosomes. We find that orthologous gene pairs, if they are located on the same chromosome in both species, tend to co-localize within a 3D vicinity of around 200–300 nm (Figure S4). While this reflects co-localization due to the overall chromosomal folding, in randomly assigned orthologies such mid-distance interactions (normalized by the genomic distance, nm/Mb) were depleted (Figure 1B, Fasano Franceschini test p value <0.001, Figures S4C and S4F). This pattern of orthologous gene pair clustering is conserved across several species (Figure S5). By comparison, the genomes of *Drosophila* and *C. elegans*, which have lost a substantial proportion of the ancient metazoan macrosyntenic signal,^12^ do not show a clear difference between observed and randomized orthologous gene pair clustering (Figures S5E and S5F).

To further investigate this result in the context of chromosomal homologies, we identified homologous chromosomes using published approaches^6^^,^^12^ (Figure S6). Previous studies have shown patterns of largely one-to-one correspondence between animal chromosomes, with individual genes retaining their chromosomal identity (yet in a scrambled order) in multiple species.^3^^,^^6^^,^^12^ Conversely, around half of orthologous genes move and disperse across multiple animal chromosomes. Genomes can thus be partitioned into genes which stay within the homologous chromosomes (ancestral linkage groups, ALGs) and those that disperse or move to other chromosomes (losing their ancestral linkage group identity, nonALG). We tested whether genes that are maintained in their ALG identity, and thus define the macrosyntenic pattern, are more likely to come into interactions with each other than dispersed genes.

We investigated contact density between ALG bins (consisting of at least one ALG gene) and nonALG bins (lacking the presence of any ALG genes) within each IntSph. Similar to orthologous gene pairs, we found that the ALG bins display more contacts than nonALG bins (consistently across several IntSphs sizes, Figure S7). Majority of ALG-ALG contacts were enriched with Euclidean distance ∼150 nm (Figures 1C, 1D, and S8), bridging ALG-ALG pairs located up to 20 Mb away from each other (in both amphioxus and blood clam). This observation is striking in the context that there were less ALG-defining genes compared to nonALG genes (altogether 5225 and 5764 ALG genes, while 8857 and 9279 nonALG orthologous genes in amphioxus and blood clam, respectively; ALGs and nonALGs occupied similar number of bins, Figures S8E and S8F), thus interactions between ALG-ALG bins should be less abundant if occurred randomly. This result was consistent when inspecting 3D chromosomal models generated using only significant interactions defined by FitHiC^11^ (Figures S8C and S8D) to avoid modeling bias as potential noise coming from Hi-C matrices. Interestingly, we find that while this pattern is overall conserved between homologous chromosomes (Figures S8A and S8B), some chromosomes stand out: in particular, chromosomes 3 and 4 in amphioxus have more nonALG-nonALG contacts (28 and 25 interacting pairs for chromosome 3 and 4, respectively) while having a very low contact density for ALG-ALG contacts (12 and 10 interacting pairs, respectively). This observation is notable as these chromosomes (amphioxus chromosome 3 and 4) have been shown to undergo recent fusions.^6^ Similar to orthologous gene pairs, the macrosyntenic pattern in *Drosophila* and *C. elegans* was different from the rest of the animals (Figure S9), highlighting that spatial signal is consistent with the evolutionary syntenic history.^12^ Together, this suggests the presence of evolutionary constraints to maintain global chromosomal organization.

**Figure 2:**
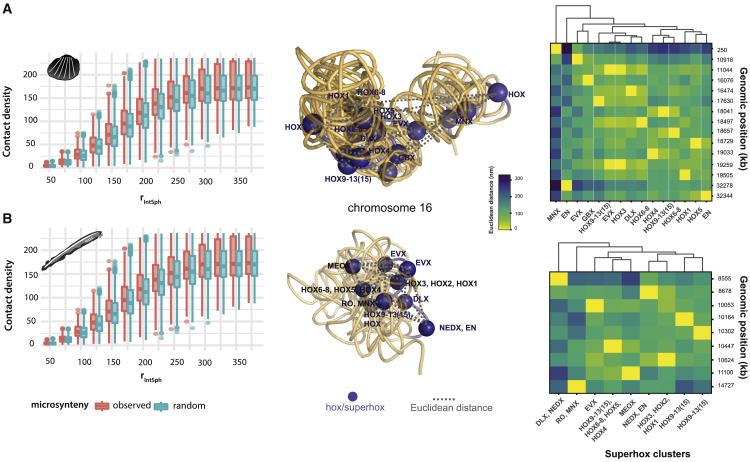
Genes in microsynteny form local 3D interaction hubs (A and B) Distribution of observed and randomly sampled microsynteny contact density [defined as number of interacting partners within IntSph of selected radius] (left) in blood clam (A) and amphioxus (B), respectively. A range of rIntSph (50–400 nm) was explored, reaching a plateau at rIntSph ∼300 nm. 3D model representation of chromosome 16 (middle panel) with hox/superhox gene clusters labeled as blue spheres. Gray dashed lines represent spatial connectivity and distances between a pair of *superhox* genes. Right panel: clustered spatial distances (columns) between superhox genes in amphioxus and blood clam against their genomic location (rows).

To further understand the properties of local genome organization, we assessed 3D contacts between known microsyntenic regions (defined here as local conserved gene clusters of 3 or more genes, allowing up to 5 intervening genes, Methods). We observed that IntSphs consisting of at least one microsynteny tend to have significantly higher number of contacts (mean 7.68 and median 6.0 interacting partners, or bins, inside IntSph) compared to IntSphs with only randomly sampled microsyntenies (clusters of genes sampled from random regions of the genome, with similar size and orthologous content as the observed microsyntenies, see^5^) (mean 6.74 and median 5.0 interacting partners, Figure 2A - Wilcoxon test p value 6.5 × 10^−4^ and 3.9 × 10^−10^ for amphioxus and blood clam, respectively). This suggests that local microsyntenic linkages can also be associated with 3D interaction hubs that go beyond the local microsyntenic vicinity and span at least the radius of the profiled interaction spheres.

### Novel spatially co-localized interactors

While little is known about the functionality of the majority of observed microsyntenic linkages, we investigated the folding around well-studied and often syntenic homeobox genes.^13^^,^^14^ The Hox cluster itself forms a tight microsynteny in most animal genomes examined to-date, yet genes from the proposed ancestral SuperHox and NK clusters^15^^,^^16^ are usually dispersed along their respective chromosomes (Figures 2B and 2C).^12^ We find that the amphioxus 3D model provides evidence for co-localization of the proposed SuperHox cluster genes within a single “sphere” of 75–100 nm, while in blood clam the genes appeared to be more dispersed (Figure 2). While this can be an artifact of the Hi-C quality, our analysis still revealed 14 orthologous genes that were located in spatial proximity (within a single 50 nm IntSph) of the proposed SuperHox cluster genes in both blood clam and amphioxus and thus could form evolutionarily conserved interacting partners (Figure 2 and S10). The region of the mouse genome spanning three of these genes (*atp5g3*, *atf2*, and the *jazf2* pseudogene) has been found in the local vicinity of and interacting with the promoters of *hoxd* genes and regulate their transcription.^17^ This contrasts with their syntenic state in amphioxus and blood clam: while the orthologs to *atp5g1/⅔*, *atf2/7*, and *jazf1/2* are located on the same chromosome as the *hox* genes in these animals, they are not in the genomic vicinity of the *hox* genes (Figure S10). Our inference of their interactions in invertebrates thus suggests importance of the retained topological, rather than local genomic, interaction around this cluster.^14^ Similarly, we can also detect six shared genes in the NK chromosomal network (Figure S10); however, their function is largely unknown. Despite the substantial gene scrambling and disruption of many microsyntenic linkages between homologous chromosomes, this analysis points to the existence of evolutionary 3D constraints upon chromosomal organization that can only be detected via 3D modeling approach.

### Outlook

We have presented an initial analysis for the comparative quantification of animal chromosome shapes and the disposition of local and chromosomal-scale gene clusters they harbor. This complements emerging cross-species analyses of topological organization^18^ with 3D organization and syntenic information. To aid the study, we developed an “interaction sphere” (IntSph) approach, which allows for approximation to compensate for Hi-C quality. Our study focuses on implementation of this method for several phylogenetically key animal clades, and paves the way for further application in other species, once supplied with higher quality Hi-C datasets. Combined with comparative genomic analyses of gene linkages at several scales of genomic organization, we show evidence for the existence of conserved 3D constraints on genome folding. In particular, we show evidence that genes within both macro- and microsyntenies display more 3D contacts than non-syntenic (and randomly sampled) regions. Our novel methodology paves the way for further detection of topologically and evolutionarily conserved genomic regions (spatiosynteny), providing testable hypotheses for their functional profiling.

### Limitations of the study

3D modeling of chromosomal topologies remains a difficult task that is highly dependent on the underlying Hi-C data quality. We used a partitioning approach to partially supplement the Hi-C quality-dependent effects on 3D genome models. Further experimental validation will be required to test the results provided here. The comparative aspect relies on accurate orthology information; for this, we tested several approaches (mutual best hit and conventional OrthoFinder approach) and assessed spatiosyntenic properties of gene pairs, micro- and macrosynteny. Our results must further be corroborated as more topological data for phylogenetically informative species become available.

## STAR★Methods

### Key resources table

**Table:** 

RESOURCE	SOURCE	IDENTIFIER
**Experimental models: Organisms/strains**		

*A. broughtonii*	Muscle tissue Hi-C	SRX5337861
*B. floridae*	whole body Hi-C	SRX3274438
*D. melanogaster*	Embryo Hi-C	SRX2947125
*C. elegans*	mixed stage embryos Hi-C	SRX2638356
*R. esculentum*	whole body Hi-C	SRX8210228
*A. fulica*	abdominal foot Hi-C	SRX5181756
*H. vulgaris*	whole polyp Hi-C	SRX14496554
*P. maximus*	Muscle Hi-C	SRX6848914

**Deposited data**		

PDB models and syntenic blocks	This manuscript	This manuscript

**Software and algorithms**		

C++	Hi-Chrom	https://github.com/TerezaClarence/Chromosome-reconstruction

### Resource availability

#### Lead contact

Further information and requests for resources should be directed to Tereza Clarence (clarence.tereza@gmail.com).

#### Materials availability

This study did not generate new materials.

### Method details

#### Orthology assignment

Genomes, annotations and protein sequences of 80 species were obtained from the databases summarised in Data S1. Only the longest isoform of each gene was retained. Orthogroups were identified using Orthofinder v2.4.1,^19^ in conjunction with diamond 0.9.36,^20^ and MCL 14.137.^21^ Identification of Hierarchical Orthogroups at the root node (root node HOGs) is based on orthogroup gene trees and species trees that were built using both Mafft 7.427^22^ and FastTree 2.1.11.^23^ For all the following analyses, we consider genes belonging to the same root node HOG to be orthologs. For ALG and pairwise ortholog comparisons we have used the annotated reciprocal best blast hit set of 6,766 orthologs from ref.^12^

#### Idenitfication of microsyntenic blocks

Microsyntenic blocks were inferred using methods described in.^5^ In order to determine which microsyntenic blocks of *A. fulica*, *Anadara broughtonii* and *B. floridae* were present in their Last Common Ancestor (Nephrozoan node), we identified blocks that were present in at least two species of each Nephorozoan ingroup or of one ingroup and the outgroup. To obtain background information, we employed two block randomization methods. For each observed block, 100 random blocks were sampled either across the whole genome, as described in ref.^5^, or only across the same chromosome bearing the observed block. All detected microsyntenic blocks, their positions and annotations are available in Data S2.

#### Macrosynteny dotplots and assignment of genes to ancestral linkage groups

Pairwise ortholog oxford plots for all possible species pairs between *A. fulica*, *A. broughtonii*, *B. floridae* and *R. esculentum* were generated, using the previously identified root node HOGs, but retaining only one-to-one orthologs for each species pair. Chromosome homologies were assessed with a Fisher exact test against a null model of gene permutation (i.e. if p < 0.05, chromosomes were considered to be homologous), with Benjamini-Hochberg correction for false discovery rate for the multiple tests done for each species pair.

Another set of ortholog oxford plots between all the possible species pairs between *A. fulica*, *A. broughtonii* and *B. floridae* were also built. Here, one-to-one orthologs were defined based on a reciprocal best hit approach, using *B. floridae* as a reference. Only the proteins of *B. floridae* with a reciprocal best hit in *A. fulica* and *A. broughtonii* were retained (4866 groups of 3 proteins each). Each group was assigned to the same BLG as the *B. floridae* protein they comprised according to ref.^6^

#### Annotation of homeodomain proteins

The homeodomain HMM profile from Pfam (http://pfam.xfam.org/family/pf00046) was used to query the proteomes of *B. floridae*, *A. broughtonii* and *A. fulica*, using hmmsearch from the HMMER 3.3 package^24^ with a 0.1 e-value threshold. Homeodomain candidates were then used to query the HomeoDB2 database^25^ using the blastp utility from the BLAST + package. If at least 8 out of the 10 top hits of a query were of the *Antennapedia*-*class* (ANTP), they were considered as ANTP candidates.

ANTP candidates were then aligned to the homeodomains of the ANTP class from HomeoDB2 using Mafft 7.427 with default parameters. The alignment was trimmed using the gappyout algorithm of trimAl^26^ and used to infer a phylogenetic tree with FastTree 2.1.11.^23^ This tree was used to isolate the *B. floridae, A. broughtonii* and *A. fulica* orthologs to the members of the SuperHox (*hox* genes, *dlx*, *en*, *evx*, *gbx*, *hhex*, *meox*, *mnx*, *nedx* and *ro*), the ParaHox (*cdx*, *gsx* and *pdx*), the NK (*nk* genes, *lbx*, *lcx*, *msx*, *tlx* and *ventx*) and NK2 cluster (*msxlx*, *nk2.1, nk2.2*) (reviewed in ref.^16^) In addition, a second round of search for orthologs was performed. To this end, all the proteins of *B. floridae*, *A. broughtonii* and *A. fulica* found in the same Orthofinder root node HOGs as the already isolated SuperHox, ParaHox, NK and NK2 cluster genes were recovered. These candidates were used as queries against the BLAST nr database. If the top hits were annotated members of the SuperHox, ParaHox, NK or NK2 clusters, they were added to the existing list of putative orthologs.

#### Hi-C analysis

Hi-C sequencing libraries were downloaded from NCBI Sequence Read Archive using SRA Toolkit.^27^ Hi-C data of *A. fulica**^28^* (accession ID SRX5181756), *B. floridae*^6^ (accession ID SRX3274438), *A. broughtonii*^29^ (accession ID SRX5337861), *P. maximus* (accession ID SRX6848914), *R. esculentum*^30^ (accession ID SRX8210228). *H. vulgaris* (accession ID SRX14496554), *C. elegans* (accession ID SRX2638356) and *D. melanogaster* (accession ID SRX2947125), together with their reference genomes were used for pre-processing (further information listed in Table). Sequence quality was evaluated using FastQC 0.11.8 (https://github.com/s-andrews/FastQC) and HiC-Pro 2.11.1 software^31^ was used to generate interaction matrices between 150 kb windows of each chromosome. Bowtie2^32^ and samtools 1.11,^33^ as a part of HiC-Pro, were utilised to map the Hi-C reads to the reference genome assemblies.

Sequencing depth was determined as (*number_paired_reads∗2∗read_length*)*/genome_size*.

Additionally, in order to find the intra-chromosomal significant interactions from our HiC-Pro results, we used FitHiC^11^ 2.0 with default parameters.

#### 3D model generation

The 3D structure of individual chromosomes was constructed using a home-built C++ software, motivated by studies *Chrom3D*^34^ and *NucDynamics*.^35^^,^^36^ Each chromosome has a beads-on-a-string representation and starts with a randomized conformation. Then, the time evolution of chromosome conformation is governed by the Newton equation of motion, with forces (detailed below) implemented to characterize the chromosome structural integrity (${\vec{F}}_{i}^{ten}$), volume exclusion between spatially overlapping genomic sites (${\vec{F}}_{i}^{rep}$), drag by nucleoplasm ($-\gamma {\vec{v}}_{i}$), and genomically distant interactions suggested by Hi-C (${\vec{F}}_{i}^{Hi-C}$).

#### The dynamics

The dynamics of a coarse-grained chromatin bead i is governed by the following Newtonian equation of motion:

$${m{\vec{a}}_{i}=-\gamma {\vec{v}}_{i}+\vec{F}}_{i}^{rep}+{\vec{F}}_{i}^{ten}+{\vec{F}}_{i}^{Hi-C}$$

where ${\vec{a}}_{i}$ and ${\vec{v}}_{i}$ are the instantaneous acceleration and velocity of the bead, respectively; m is the mass of the bead; γ is the drag coefficient; ${\vec{F}}_{i}^{ref}$, ${\vec{F}}_{i}^{ten}$, and ${\vec{F}}_{i}^{Hi-C}$ are forces implemented in the model to characterise the mutual volume exclusion between beads, the interaction between genomically consecutive beads, and the interaction between genomically distant beads with high Hi-C frequency. Computationally, Verlet integration is applied to calculate the trajectories of chromosome beads over time.

#### The volume exclusion force

The volume exclusion between any two spatially overlapping beads is assumed linearly elastic. The contribution of this force to a bead i is described by the following equation:

$${\vec{F}}_{i}^{rep}=\sum_{j\neq i}^{N}K^{rep}\left(d_{i,j}-d_{rep0}\right){\hat{u}}_{i,j},ifd_{i,j}<d_{rep0}\text{,}$$

where $K^{rep}$ is the spring constant reflecting the incompressibility of genetic content within the beads in contact; $d_{i,j}$ is the distance between the center of two consecutively connected beads *i* and *j*; $d_{rep0}$ is the rest length of the linearly elastic spring (in our case $d_{rep0}\;2*r_{bead}$); ${\hat{u}}_{i,j}$ is a unit vector pointing from bead i to bead j.

#### The chromatin tension force

The interaction between two genomically consecutive beads is assumed to be linearly elastic. The contribution of this force to a bead i is described by the following equation:

$${\vec{F}}_{i}^{ten}=K^{ten}\left(d_{i,i-1}-c_{2}\right){\hat{u}}_{i,i-1}+K^{ten}\left(d_{i,i+1}-c_{2}\right){\hat{u}}_{i,i+1}$$

where $K^{ten}$ is the spring constant of the inter-bead 'chromatin' linker, $d_{i,i+1}$ is the distance between the center of two consecutively connected beads i and i+1; $c_{2}$ is the rest length of the linearly elastic spring; ${\hat{u}}_{i,i+1}$ is a unit vector pointing from bead i to bead i+1.

#### The Hi-C restraint force

The interaction between genomically distant beads is also assumed to be linearly elastic. The contribution of this force to a bead i is described by the following equation:

$${\vec{F}}_{i}^{Hi-C}=\sum_{j\neq i}^{M}K^{Hi-C}\left(d_{i,j}-d_{Hi-C0}\right){\hat{u}}_{i,j},ifp_{i,j}>p_{rep0}$$

where $K^{Hi-C}$ is a constant reflective of the constraint strength implied by Hi-C and applies to any pairs of coarse-grained beads that have pairwise Hi-C frequency greater than a threshold value, namely, $p_{i,j}>p_{rep0}$; $d_{Hi-C0}$ is the rest length of the linearly elastic spring; ${\hat{u}}_{i,j}$ is a unit vector pointing from bead i to bead j.

Specific values for $K^{rep}$, $K^{ten}$ and $K^{Hi-C}$ were determined based on parametrization obtained the chromatin condensation tool reported in our previous study *Gerguri et al.*^37^

#### Data preparation and modeling

Normalised HiC-Pro sparse matrix was parsed into matrices of separate chromosomes, containing *cis* interactions only. Hi-Chrom, similarly to 3DMax^10^ or LorGD,^9^ has an option to set an IF (interaction frequency) cutoff to filter out interactions which should be then utilised as modeling constraints. One could also feed Hi-Chrom with an already pre-filtered Hi-C matrix where all the interactions would be used for modeling. For the purpose of this study, we reconstructed single chromosome models using only *cis* interactions due to the quality of utilized Hi-C where *trans* interactions are largely absent or could be biased due to not only multi-cellular but ‘multi-tissue’ nature of the data. The reason for setting up a specific IF threshold for modeling constraints is that high number of contact restraints results in very dense and compact chromatin models. Such structures might be lacking desired biological relevance, however there is no current knowledge about optimal number of contacts per selected genomic region, therefore we used a mean value of interaction frequency as a Hi-C cut-off to filter out interactions used for modeling. The total number of *cis*-contacts per chromosome together with the number of used constraints for reconstruction with different Hi-C threshold is shown in Tables for amphioxus and blood clam, respectively. Structural measurements of mapped syntenic blocks (such as SASA, coverage, depth) can be, to some extent, affected by the number of constraints and thus compactness of chromatin model. We keep the selection criteria for Hi-C threshold consistent among individual chromosomes to mitigate this impact. We reconstructed chromosomal scaffolds of blood clam and amphioxus with different interaction frequency (IF) cut-off thresholds (see species tables below) and compared the models. Overall, the geometry and fine topology of the models was very similar between different IF cut-off thresholds (data not shown). The higher the number of constraints utilised to build a model, the more densely packed the chromosomal scaffold was. Since the quality of the Hi-C datasets utilised is variable (Table), we selected models from mean IF cut-off to be further analyzed, in order to capture the majority of interactions obtained from Hi-C, along with models generated from significant *cis* interactions obtained via FitHiC tool.^11^ All other species were reconstructed using mean IF values (for *cis* interactions) as a threshold.

All chromosomes were reconstructed with three replicates and each model was initialised with different conformation based on principles of self-avoiding random walk (SAWR). The reconstruction process starts after initialization, when the pseudo-energy of 3D chromosome conformations, calculated as the sum of kinetic and potential energy in the system, is monitored throughout the simulation as an indicator of convergence of the system. This is then accompanied by root-mean-square (RMSD) analysis across all the time-point structures toward the final structure. We ran the reconstruction algorithm for 10,000-time steps and the final chromosome structure of each replicate run was then taken for further analysis. In order to validate correlation of our models with Hi-C IF map, we calculated cosine similarity along with Spearman correlation between IF contacts, which were selected as restraints for 3D modeling, and Euclidean distance of corresponding genomic position in the model (Figures S1A and S1B). Performance of Hi-Chrom was benchmarked against LorGD^9^ and 3DMax^10^ chromosome modeling tools (Figure S1). Modeling ability of Hi-Chrom was also benchmarked with distance measurement of interphase and mitotic fission yeast chromosomes as shown in *Gerguri et al*.^37^ Major motivation for Hi-Chrom development was to alleviate problems with parallelization of previous modeling tools and dependency complications with computational clusters.

### Quantification and statistical analysis

#### Model analysis and gene mapping

Genes and microsynteny locations were mapped onto the chromosome models within the 150 kb-resolution beads. Due to low resolution, some 150 kb-regions include multiple ALGs and nonALGs together. If at least one ALG is present per 150 kb-bead, we treat this region as ALGs content only.

#### Interaction sphere (IntSph) analysis

In order to identify spatio-functional units within chromosome scaffolds, we performed sliding Interaction Sphere (IntSph) analysis; an imaginary sphere with specific radius moving along the chromosome scaffold and detecting spatial contacts within (Figures 1A, S2A, and S2B).

The smaller the radius of IntSph, the more of the local genomic contacts are dominant as interacting partners within IntSph. The larger the radius, the more genomically long-range interactions can be included as demonstrated in Figure S2A. To measure the IntSph occupancy we defined ‘*contact density’* as the number of interacting beads within a defined radius of IntSph. The suggestion for optimal selection of r_IntSph_ is to first explore a broad range of values based on mean and maximum Euclidean distances of chromosome models to be analyzed; the distribution of contact density for selected r_IntSph_ should follow sigmoidal fit. We suggest touse 0.5-1.0∗inflection point value for optimal r_IntSph_ depending on biological question.

#### Model visualisation

3D models were outputted in modified PDB format and visualised using PyMOL.^38^

## Data Availability

The 3D modeling software Hi-Chrom, utilised in this study to model chromosome scaffolds, is available for free use at location: https://github.com/FrancisCrickInstitute/Chromosome_modelling and https://github.com/TerezaClarence/Chromosome-reconstruction, along with PDB files of chromosome models for each species.

## Supplemental information

Document S1. Figures S1–S10

Data S1. Species used in orthology construction and their abbreviations, related to STAR Methods

Data S2. Microsynteny locations and annotation table, related to STAR Methods
