# Supplementary material for: Robust 3D modeling reveals spatiosyntenic properties of animal genomes
Source: iScience. 2023 Feb 4;26(3):106136. doi: 10.1016/j.isci.2023.106136 (PMC9976460; doi:10.1016/j.isci.2023.106136)
Supplement: Document S1. Figures S1–S10 [file mmc1.pdf]

iScience, Volume *26*

Supplemental information

Robust 3D modeling reveals spatiosyntenic properties of animal genomes

Tereza Clarence, Nicolas S. M. Robert, Fatih Sarigol, Xiao Fu, Paul A. Bates, and Oleg Simakov

Supplementary Items

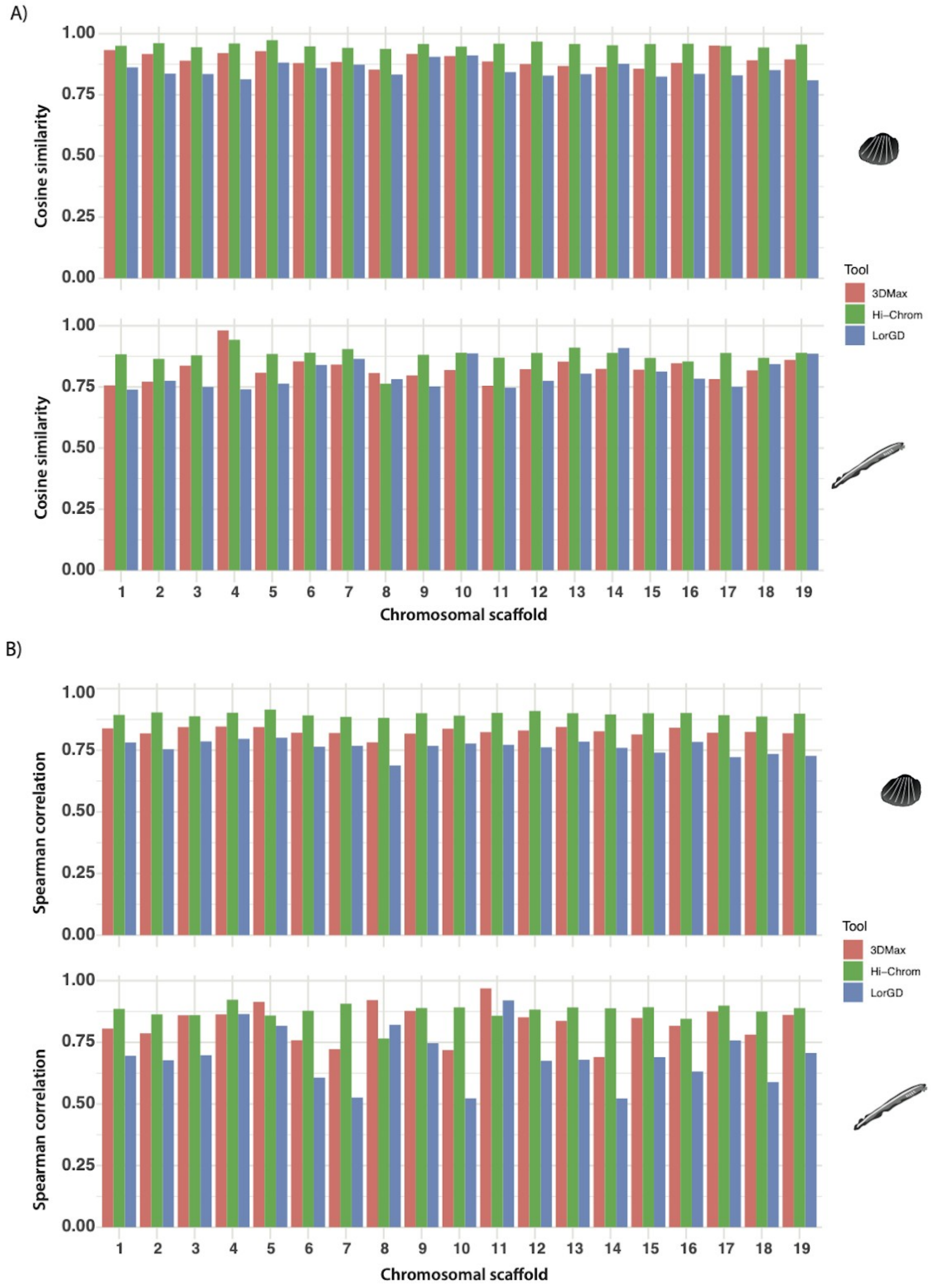

Supplementary Figure 1. Modelling validation. Related to Figure 1. Three-dimensional chromosome models (prepared using Hi-Chrom) were validated against models generated from LorGD and 3DMax. Cosine similarity (A) along with Spearman correlation (B) were used to assess the model quality.

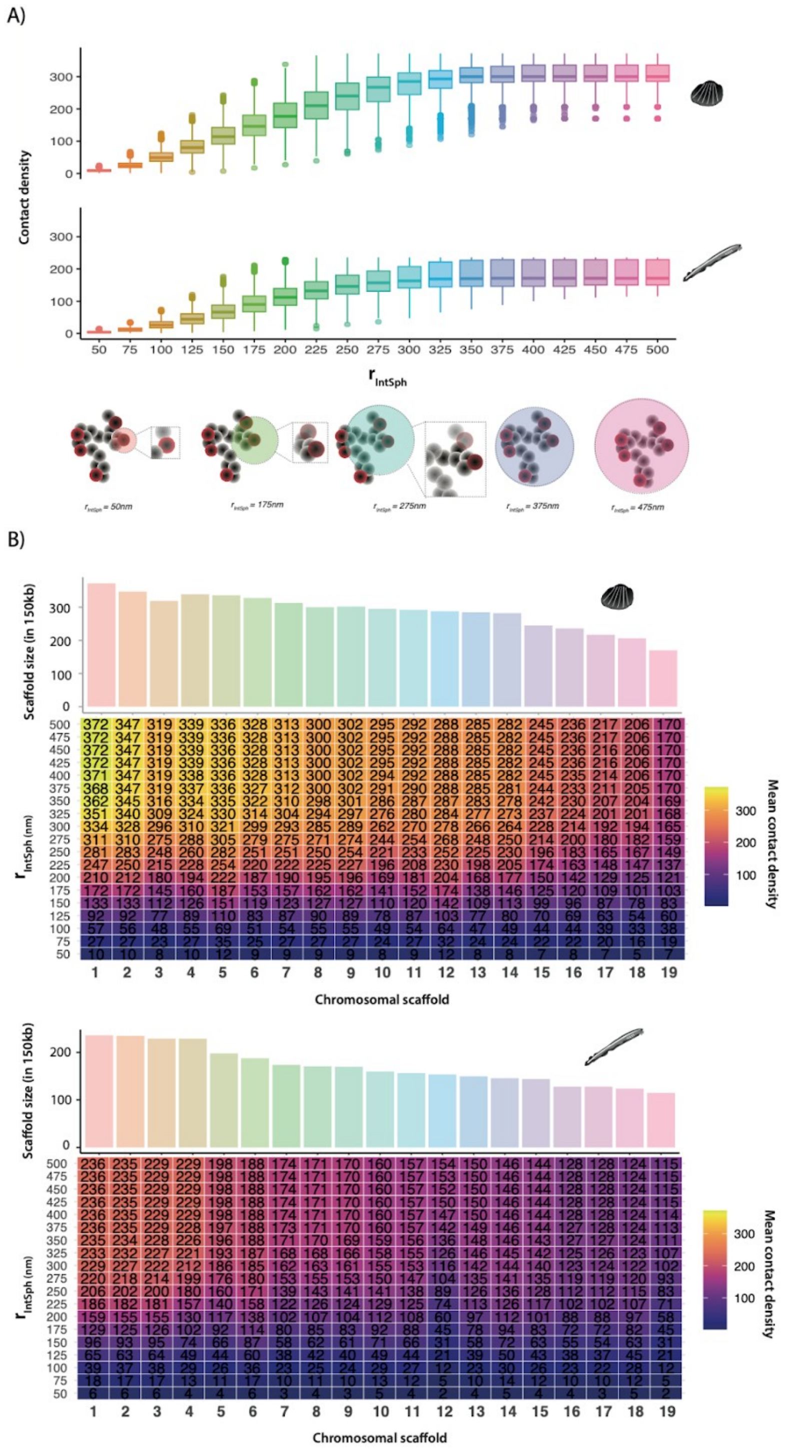

Supplementary Figure 2. Interaction profiling effectively reflects on chromosome geometry. Related to Figure 1. A) Number of interactors present within IntSph of selected radii shown as distributions across a wide range of r_{IntSph} (50-500nm) for blood clam (upper panel) and amphioxus (lower panel). Schematic below the graph demonstrates analysis of 'sliding IntSph with variable number of interactors captured within selected radius of IntSph'. B) Mean number of interactors per chromosome scaffold in blood clam (upper panel) and amphioxus (lower panel) for selected r_{IntSph}. Bar plots above heat maps show the relative size of each chromosome scaffold in 150kb-bins. Such interaction profiling of chromosome models on a per-scaffold basis allows to select appropriate radius of IntSph independently for each chromosome to achieve meaningful results.

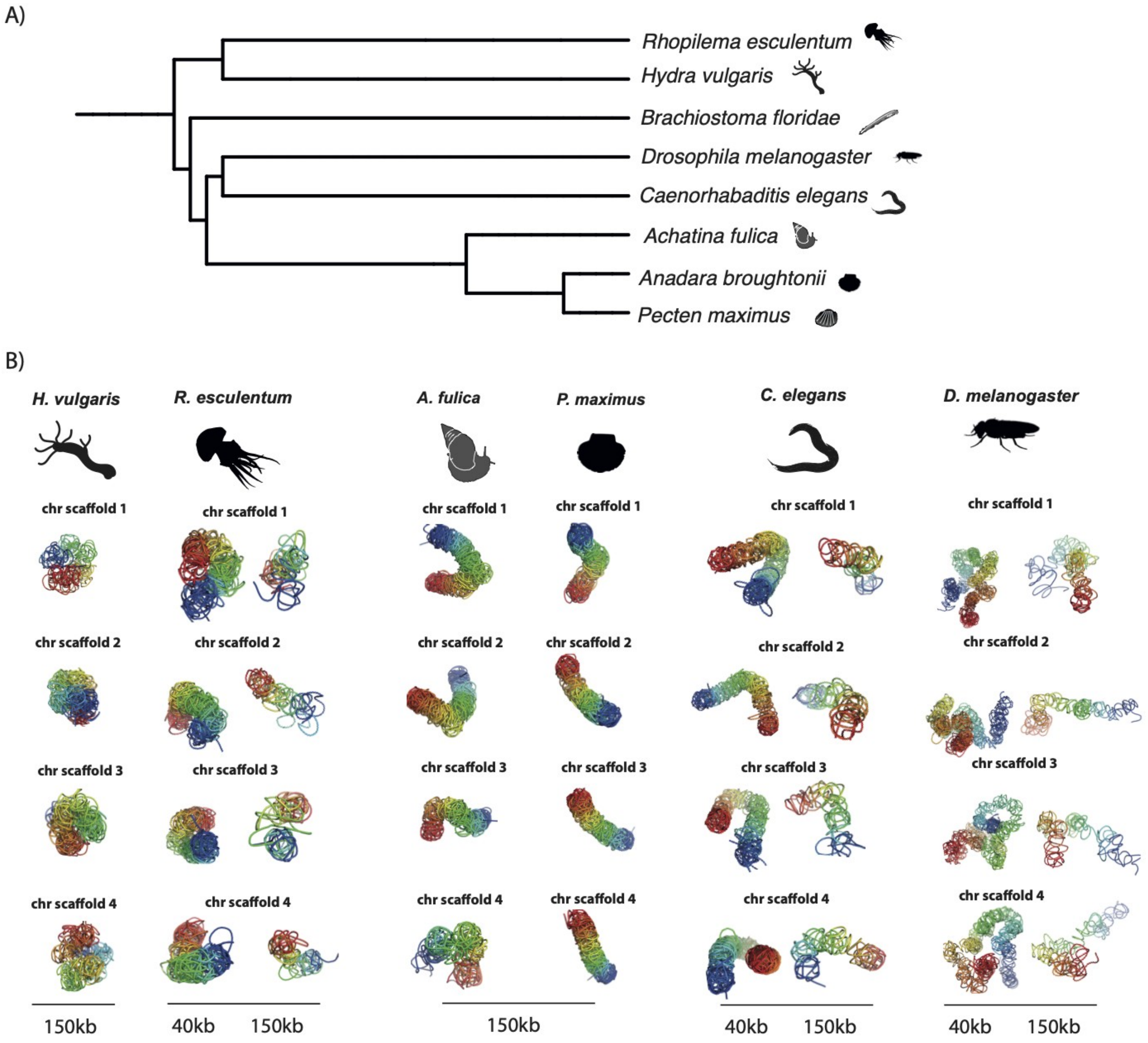

Supplementary Figure 3. Examples of chromosomal models for selected species. Related to Figure 1. Cladogram (via iTOL online) depicting evolutionary relationships for species utilised in this study (A). Examples of 4 largest chromosomal scaffolds for 150kb-bin and for 40kb-bin resolution (B). 3D models of chromosomal scaffolds are colour-coded according to the position along the chromosome.

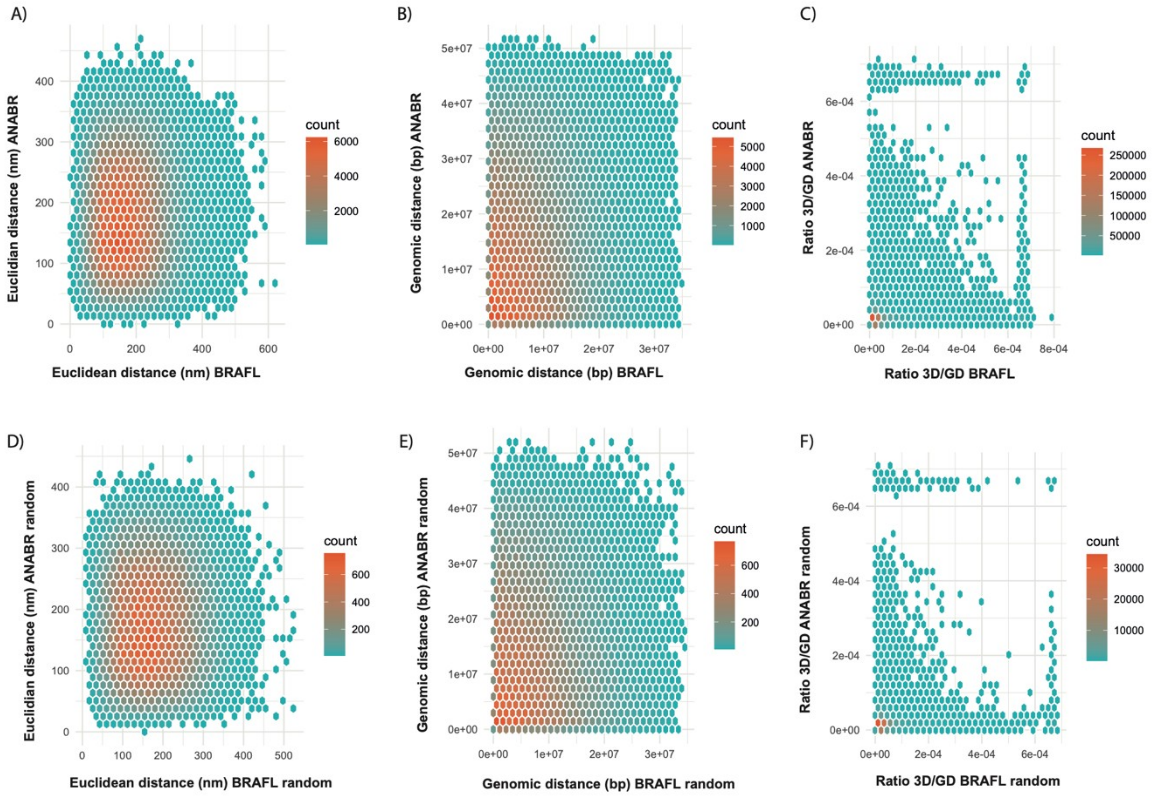

Supplementary Figure 4. The relationship between genomic and Euclidean distances in the sets of observed and randomised ortholog gene pairs. Related to Figure 1. Only orthologous gene pairs that map to the same set of chromosomes were considered. A,D) Euclidean distances between ortholog pairs in observed and fully randomised orthology set, respectively, reflecting the overall shape of the chromosomes; B,E) Genomic distances between ortholog pairs in observed and fully randomised orthology set, respectively; C,F) Ratios between Euclidean and Genomic distances between orthologous gene pairs in observed and randomised orthologies, respectively. Observed orthologous gene pairs are more commonly found in the 20 - 40 nm/Mb region, indicating that more distant interactions between orthologs are present in the observed set. Zero distance (within the same bin) interactors were excluded.

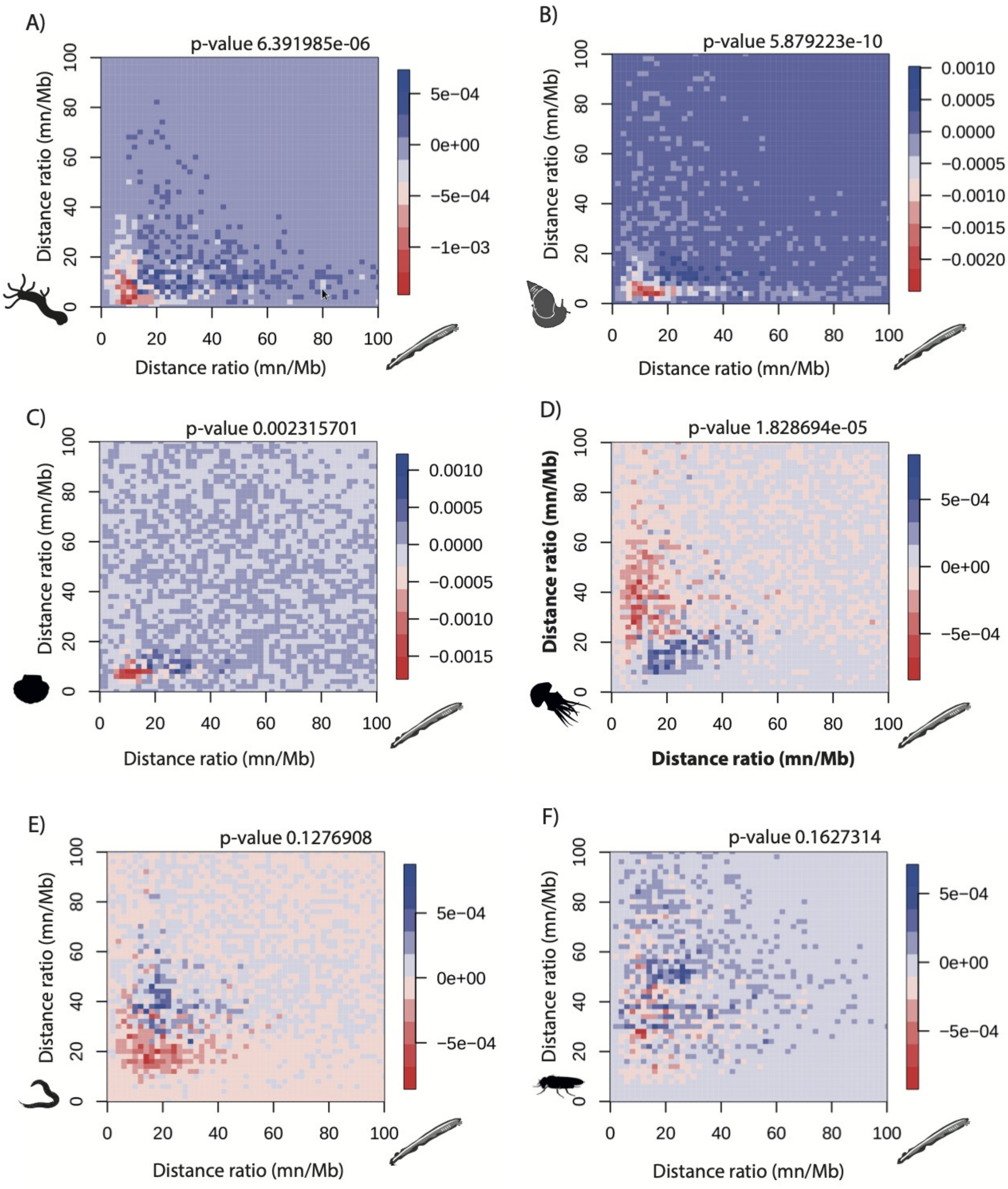

Supplementary Figure 5. Enrichment of spatio-genomic signal in observed compared to randomised orthologous gene pairs. Related to Figure 1. Difference between observed and randomised spatiosyntenic ratios (Euclidean relative to genomic distance, nm/Mb) in various taxa: (A) Amphioxus - *Hydra vulgaris*, (B) Amphioxus - African snail *Achatina fulica*, (C) Amphioxus - scallop *Pecten maximus*, (D) Amphioxus - jellyfish *Rhopilema esculentum*, (E) Amphioxus - the nematode *Caenorhabditis elegans*, (F) Amphioxus - *Drosophila melanogaster*. P-values were calculated as a median of 10 independent Fasano Franceschini tests (Bonferonni-corrected) for each species pair.

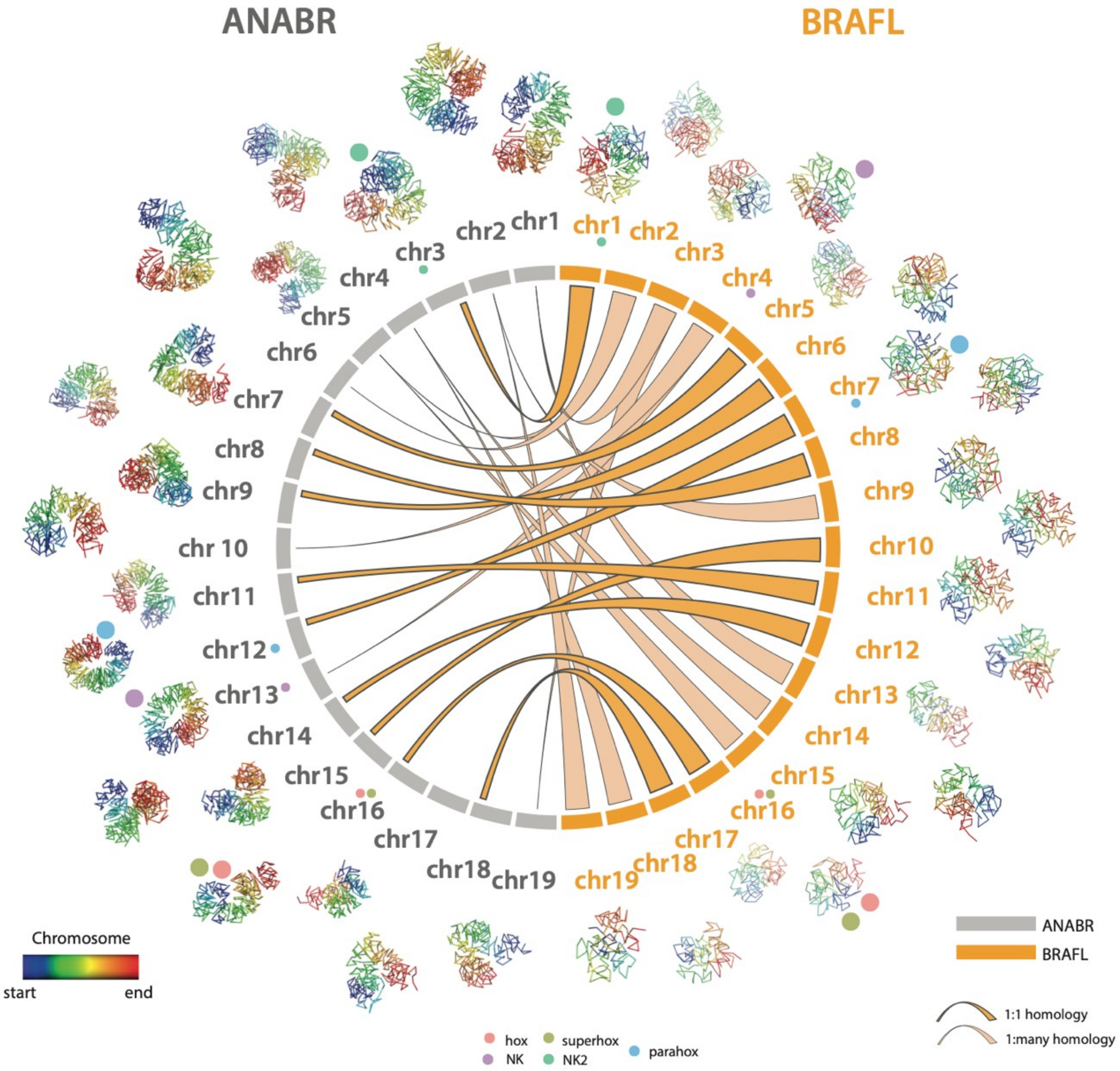

Supplementary Figure 6. 3D models of chromosomal scaffolds in amphioxus and blood clam. Related to Figure 1. Circos plot depicting homology between chromosomes of amphioxus (BRAFL) and blood clam (ANABR). Dark orange links correspond to 1:1 homology whilst light orange links show 1:many homology. The homology of two chromosomes is given when the number of shared one-to-one orthologues is greater than expected under a null model of random gene distribution ($p < 0.05$, assessed by a Fisher's exact test). The p-values were corrected using the Benjamini-Hochberg method for every chromosome-chromosome pair.

3D models of chromosomal scaffolds surround the circus plots; with each chromosome colour-coded according to the within-chromosomal nucleotide position. 3D models of chromosomal scaffolds in blood clam adopt worm-like structures with bendings and twists present, while Amphioxus models show more globular, bundle-like structures. Small circles next to the chromosomes indicate the presence of clusters of interest.

A)

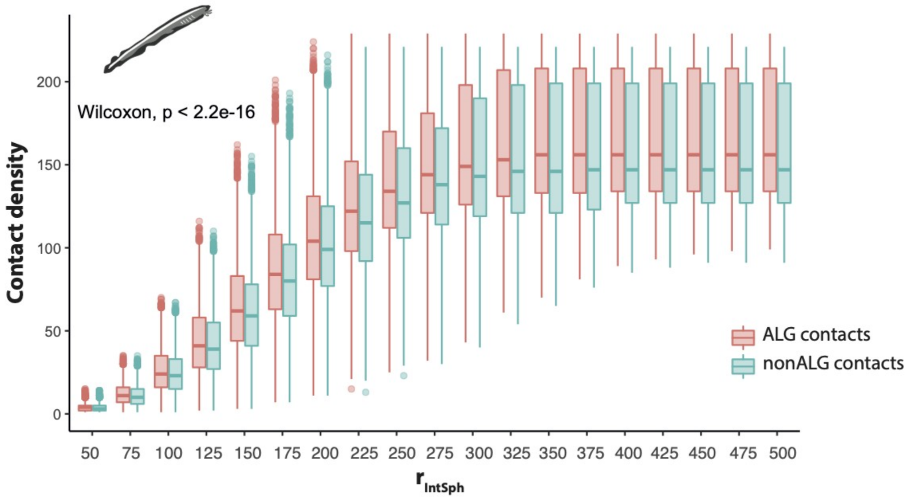

B)

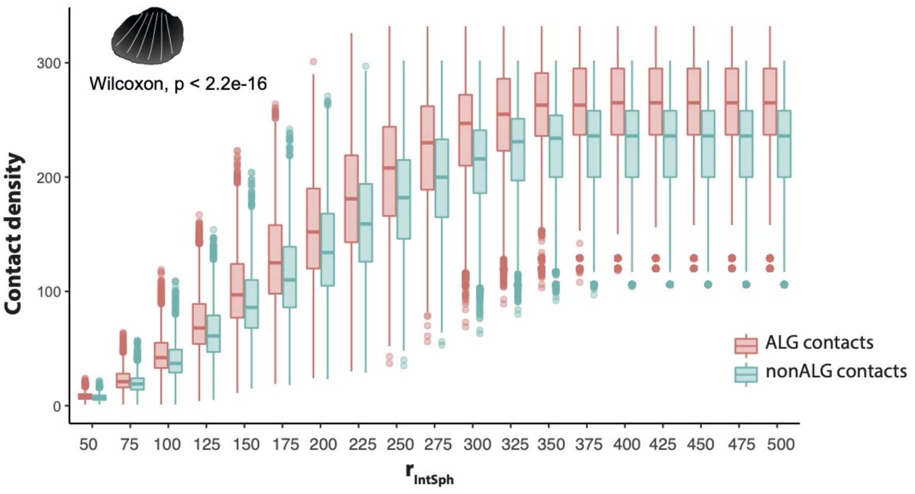

Supplementary Figure 7. Distribution of contact densities for ALG and nonALG bins at different IntSph sizes. Related to Figure 1. Contact density within ALG and nonALG IntSph for a broad range of radii was measured for amphioxus (A) and blood clam (B), respectively. Corresponding p-value calculated as a mean of all p-values for each r_{IntSph} using Wilcoxon rank sum test, showing significant difference between the number of interactions mediated by ALGs versus nonALGs for both species.

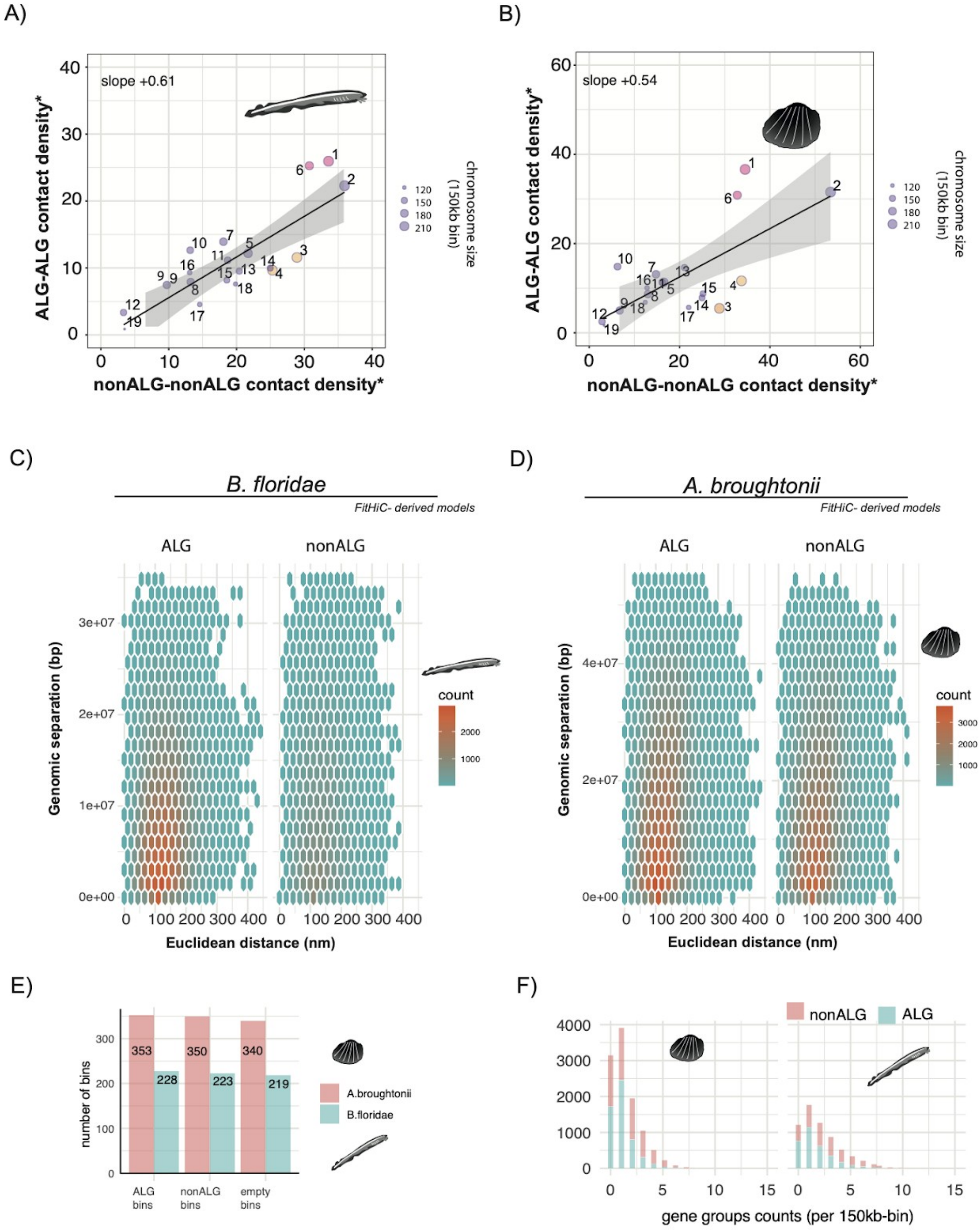

Supplementary Figure 8. Macrosynteny conservation in 3D. Related to Figure 1. A, B) Mean contact density of ALG-ALG vs. nonALG-nonALG interaction pairs within IntSph (r_{IntSph} = 50 nm) per chromosome (*normalised towards the number of ALG/nonALGs bins per chromosome) for amphioxus and blood clam, respectively. Interestingly, chromosomes 1 and 6 (pink) have more than the average ALG-ALG contacts while chromosomes 3 and 4 (orange) contain more than the average nonALG-nonALG contacts. C,D) Hexbin plot depicting distribution relationship between genomic separation and Euclidean distance of interacting ALG-ALG or nonALG-nonALG pairs from models generated using significant *cis* contacts detected via FitHiC. Both distributions from amphioxus and blood clam share the same trend as results observed on amphioxus and blood clam models generated from *cis*

interactions based on interaction frequency cutoff [selected as the mean interaction frequency value observed in Hi-C map], Figure 2C. E) Total number of ALG, nonALG and empty (depleted of both ALGs and nonALGs) 150kb-bins observed for amphioxus (light cyan) and blood clam (coral) genome, respectively. F) Number of ALGs (light cyan) or nonALGs (coral) per 150kb bin depicted as histogram for blood clam (left) and amphioxus (right), respectively.

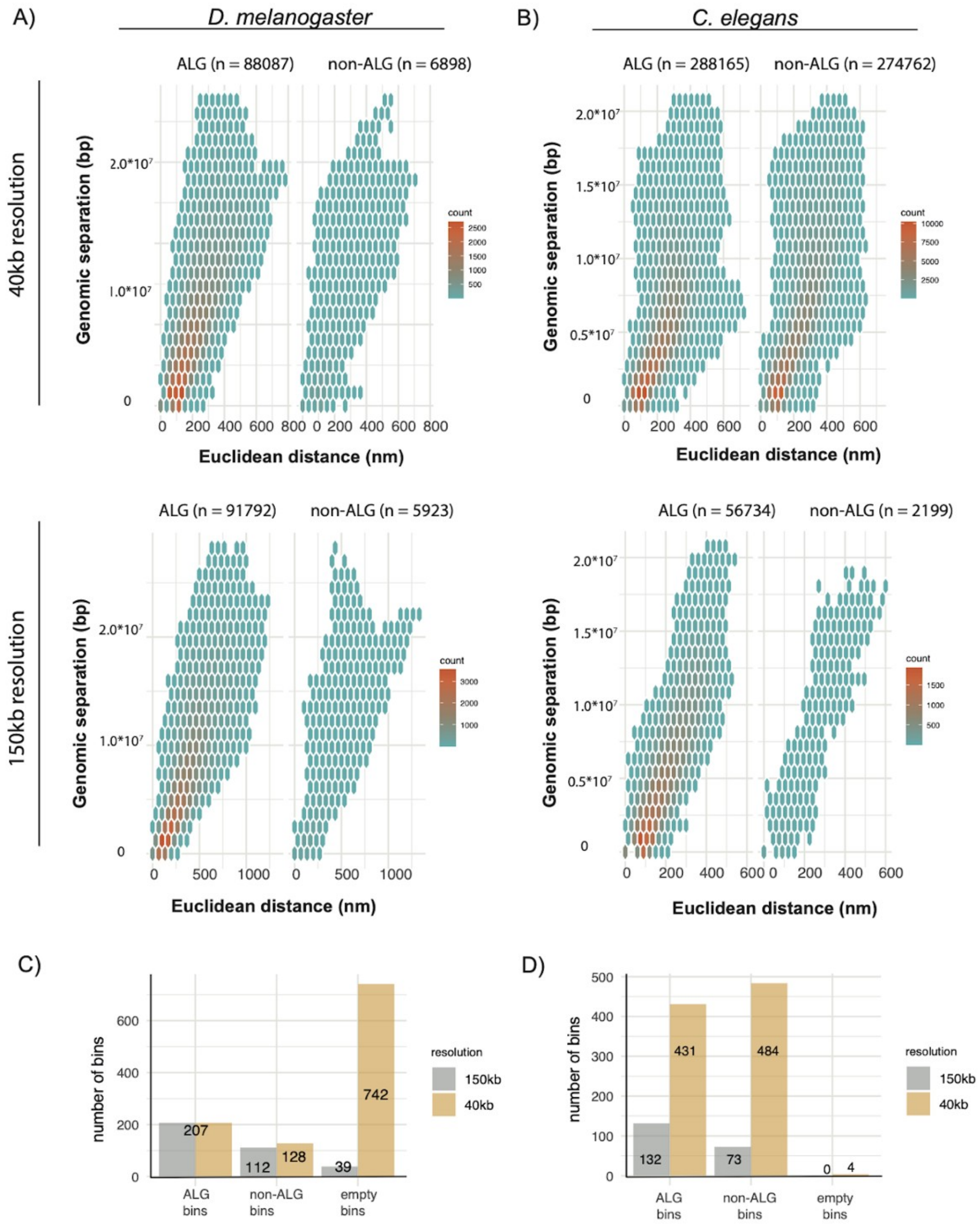

Supplementary Figure 9. Interaction pattern of ALGs in *D.melanogaster* and *C. elegans.* Related to Figure 1. A,B) Hexbin plot depicting distribution relationship between genomic separation and Euclidean distance of interacting ALG-ALG or nonALG-nonALG pairs from 3D chromosomal models of *D.melanogaster* and *C.elegans* with 40kb-bin (upper panels) and 150kb-bin (lower panel) resolution. C,D) Number of ALG-/nonALG and empty bins in 40kb (light yellow) and 150kb (light grey) models of *D.melanogaster* and *C.elegans*, respectively.

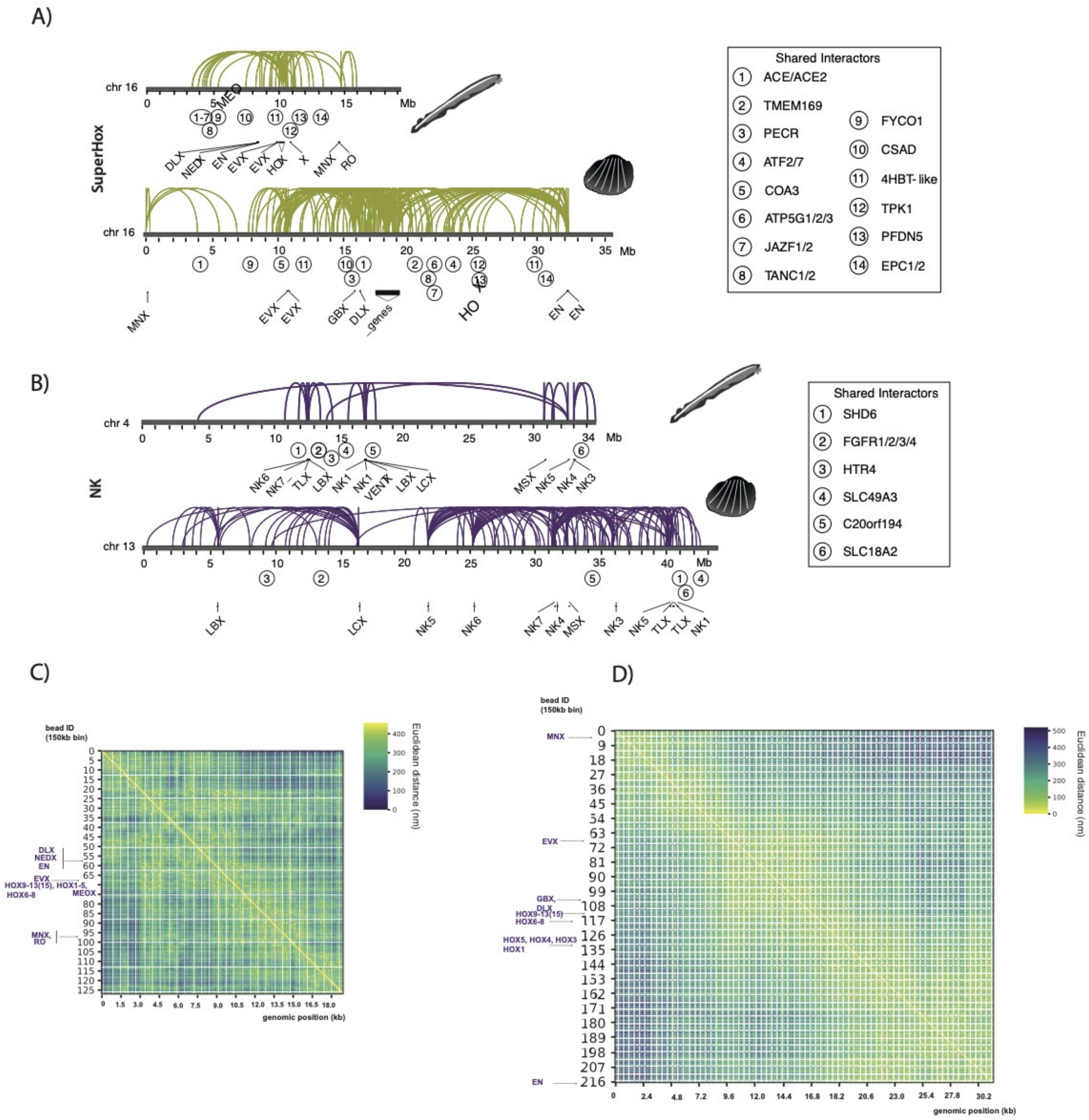

Supplementary Figure 10. Spatial colocalization of hox/superhox gene clusters. Related to Figure 2. (A, B) Interaction network of SuperHox (green, A) and NK (purple, B) clusters in amphioxus and blood clam, respectively. Circled numbers correspond to putative shared interactors of SuperHox/NK clusters between amphioxus and blood clam. (C, D) Euclidean distance matrix for chr16 in amphioxus (C) and blood clam (D) with labeled locations of superhox clusters.
